# Current Advances in Molecular Basis and Mechanisms Regulating Leaf Morphology in Rice

**DOI:** 10.3389/fpls.2018.01528

**Published:** 2018-10-23

**Authors:** Peizhou Xu, Asif Ali, Baolin Han, Xianjun Wu

**Affiliations:** ^1^Rice Research Institute, Sichuan Agricultural University, Chengdu, China; ^2^Key Laboratory of Southwest Crop Genetic Resources and Genetic Improvement, Ministry of Education, Chengdu, China

**Keywords:** bulliform cells, cell proliferation, cell wall integrity, homeostasis, phytohormones

## Abstract

Yield is majorly affected by photosynthetic efficiency. Leaves are essential structure for photosynthesis and their morphology especially size and shape in a plant canopy can affect the rate of transpiration, carbon fixation and photosynthesis. Leaf rolling and size are considered key agronomic traits in plant architecture that can subsidize yield parameters. In last era, a number of genes controlling leaf morphology have been molecularly characterized. Despite of several findings, our understanding toward molecular mechanism of leaf rolling and size are under-developed. Here, we proposed a model to apprehend the physiological basis of different genes organized in a complex fashion and govern the final phenotype of leaf morphology. According to this leaf rolling is mainly controlled by regulation of bulliform cells by *SRL1, ROC5, OsRRK1, SLL2, CLD1, OsZHD1/2*, and *NRL1*, structure and processes of sclerenchyma cells by *SLL1* and *SRL2*, leaf polarity by *ADL1, RFS* and cuticle formation *by CFL1*, and *CLD1*. Many of above mentioned and several other genes interact in a complex manner in order to sustain cellular integrity and homeostasis for optimum leaf rolling. While, leaf size is synchronized by multifarious interaction of *PLA1, PLA2, OsGASR1*, and *OsEXPA8* in cell division, *NAL1, NAL9, NRL1, NRL2* in regulation of number of veins, *OsCOW1, OsPIN1, OsARF19, OsOFP2, D1* and *GID* in regulation of phytohormones and *HDT702* in epigenetic aspects. In this review, we curtailed recent advances engrossing regulation and functions of those genes that directly or indirectly can distress leaf rolling or size by encoding different types of proteins and genic expression. Moreover, this effort could be used further to develop comprehensive learning and directing our molecular breeding of rice.

## Introduction

Rice is a model plant of monocots and an important crop that feeds more than half of the population around the globe. According to estimates, food supply will be insufficient as growth rate of population and increase in yield is not harmonious (Zhu et al., 2010). Yield is a complex quantitative trait determined by various agronomic factors of plant morphology and environment. With recent advances made in the field of molecular biology, genes and QTLs for grain yield have been identified and characterized functionally. Incorporation of those genes could into local cultivars can assist molecular breeding for improvement of yield in rice (Lafitte et al., 2004; Liu et al., 2008).

The leaf is a major photosynthetic organ in plants and its morphology such as size and rolling are key components in plant architecture that significantly affect crop yield (Walter et al., 2009). Leaf rolling is normally caused in plant species due to water scarcity (Chandra and Dubey, 2009). However, other abiotic stress factors such as temperature, salt, UV radiation and heavy metals also affect leaf morphology and yield (Subashri et al., 2009). Morphology and physiology of leaf can affect light interception, carbon fixation, photosynthetic rate, transpiration and storage assimilation (Yuan et al., 2015). Leaf rolling is a protective mechanism to avoid photo damage in plants (Nar et al., 2009; Kadioglu et al., 2012). The leaf rolling reduces the exposed leaf area to sunlight, and it is regulated by complex mechanism of several interacting genes (Kadioglu et al., 2012).

The ideotype breeding of ‘Super rice’ suggests that uppermost three leaves should be erect, long, rolled (V-shaped), narrow, and dark green in color (Zhang et al., 2009). Engineering of molecular breeding based on ideotype could be a helpful approach to increase yield (Yang et al., 2014). A long and thick leaf contains more abundant photosynthetic pigment and content of nitrogen assimilates (Ghiglione et al., 2008). Moreover, thick leaf has low tendency to bend down as it has more potential to stay erect (Nelson et al., 2002). Appropriate leaf rolling is beneficial in rice to enhance its erectness and optimum leaf angle helps in proper light interception (Yuan et al., 2015). Morphological diversity in leaf folding (inward or outward) also allows adjustments for efficient photosynthetic activity per unit leaf area (Yuan et al., 2015). Optimum leaf rolling not only enhances the accumulation of dry matter but also slows down transpiration rate by reducing absorbance of solar radiation on leaves (Lang et al., 2004). Previous studies revealed low water potential and decreased turgidity in bulliform cells cause leaf rolling (Price et al., 1997). Partial leaf rolling is effective for better water use efficiency than fully flattened or extremely curved leaf on adaxial or abaxial side (Juarez et al., 2004). To date, a large number of genes are reported that regulate leaf morphology affecting factors especially leaf rolling and size. Our review will present a comprehensive understanding about the function of these genes.

## Role of Genes in Optimum Leaf Rolling

Advancements in molecular methodologies lead to discovery of several leaf rolling genes that have been cloned and functionally characterized in rice. We have categorized those genes based on their part, which they play in final appearance of leaf.

### Leaf Polarity (Adaxial/Abaxial)

Adaxial–abaxial determinants control leaf polarity in developing leaves of plant (Yamaguchi et al., 2012). Leaf morphogenesis is an important feature of plants, as they need to establish proximodistal, mediolateral, and adaxial–abaxial axes during leaf development (Hibara et al., 2009). Manipulation in molecular breeding can modify the adaxial–abaxial axis for adjustment in organogenesis. Leaf polarity is characterized in two stages, i.e., meristem-dependent and -independent stage (Toriba et al., 2011). Adaxial–abaxial polarity is considered to have an important effect on leaf rolling. Rice *ADL1 (ADAXIALIZED LEAF 1)* gene was isolated from *adl1* mutant using positional cloning, and it encodes a CALPAIN-LIKE CYSTEINE PROTEINASE. Deficiency of CALPAIN-LIKE CYSTEINE PROTEINASE leads to rolling of leaves abaxially. Morphological analysis revealed bulliform cells specifically appear only on the adaxial side of leaf normally were also found on the abaxial side in *adl1* mutant (Hibara et al., 2009). The faulty establishment of adaxial–abaxial polarity might be responsible for the rolling of leaf. Additionally, loss of a transcription factor, *SLL1 (SHALLOT-LIKE 1)* and overexpression of *OsAGO7 (ARGONATUE)* caused leaf rolling (Shi et al., 2007; Zhang et al., 2009). A crucial gene *ROLLED FINE STRIPED (RFS)* also showed leaf rolling phenotypes in rice due to poor development of vascular bundles on adaxial side of leaf. Knock down of key elements of CHD3/Mi-2 (chromatin remodeling factor of *RFS*) caused severe leaf rolling in *rfs-1* mutant (Cho et al., 2018).

### Bulliform Cells (Number and Size)

Many genes regulate leaf rolling by affecting cytological architecture of leaf cells, e.g., bulliforms, cuticle of leaves, and sclerenchymatous cells. Bulliform cells are located in ridges of vascular bundle near midrib on the adaxial epidermis of leaf (Li et al., 2010). These are highly vacuolated and play an important role in leaf rolling by regulating their number and size^1^. Thus far, dozens of genes modulating bulliform cell number and size have been functionally characterized. *SRL1* (*SEMI ROLLED LEAF 1*) encodes a putative GPI (GLYCOSYLPHOSPHATIDYLINOSITOL) anchored protein in rice that is located in plasma membrane. The loss-of-function mutant of *SRL1* exhibits rolling of leaves adaxially due to an augmented number of bulliform cells on adaxial surface of leaf. Further studies demonstrated *SRL1* negatively normalizes the expression of genes encoding vacuolar H^+^-PYROPHOSPHATASE and H^+^-ATPASE that usually impede the development of bulliform cells (Xiang et al., 2012). Transgenic plants overexpressing *OsHox32* that belongs to *HD-ZIPIII (HOMEODOMAIN LEUCINE ZIPPER)* gene family had a similar phenotype to that of loss-of-function mutant of *SRL1*. However, besides increasing, reduced number of bulliform cells are also responsible for leaf rolling (Li Y.Y. et al., 2016).

*Curled leaf and dwarf 1 (cld1)* mutant displayed defects of leaf rolling and is allelic to *SRL1*. *CLD1/SRL1* encodes a GPI anchored protein that plays its role in formation of cell wall. The loss-of-function mutant of *CLD1/SRL1* showed lesser contents of lignin and cellulose in epidermis of bulliform cells. Defects in cell wall formation cause more rapid water loss and reduce water retaining capacity of leaves (Li et al., 2017).

Compared to adaxial rolling of leaves, suppression of *YAB1*, a member of *YABBY* gene family leads to a higher number of bulliform cells and showed abaxial rolling of leaf (Dai et al., 2007). Likewise, overexpression of *OsZHD1 (ZINC FINGER HOMEODOMAIN 1)* or its homolog *OsZHD2* and a defect in *LC2 (LEAF INCLINATION 2)* induced more number of bulliform cells that causes abaxial rolling of leaves (Zhao et al., 2010; Xu et al., 2014). Some genes control leaf rolling via regulating size of bulliform cells. Characterization of a *hal1(half-pipe-like leaf 1)* mutant in rice exhibited adaxial curling of leaves phenotype due to underdeveloped size of bulliform cells. Mutation in *hal1* mutant also affected size of leaf blade and spikelet (Matsumoto et al., 2017). *RL14 (ROLLING LEAF 14)* encodes a *2OG-Fe* (OXYGENASE PROTEIN) that is convoluted in formation of secondary cell wall of leaf (Fang et al., 2012). *NRL1 (NARROW AND ROLLED LEAF 1)* (Hu et al., 2010), encodes a CELLULOSE SYNTHASE-LIKE PROTEIN that is essential for normal biosynthesis of cell-wall (Fang et al., 2012). *OsMYB103L*, a *R2R3MYB* transcription factor usually targets *CESA* genes that are involved in regulation of cellulose synthesis (Fang et al., 2012; Yafeng et al., 2015). Transgenic plants with reduced expression level of *RL14* or *NRL1*, or overexpression of *OsMYBL103L* displayed adaxial rolling of leaves due to reduced size of bulliform cells. Moreover, all of these genes were involved in cell-wall formation, suggesting these genes may contribute to cell division and ultimately regulate size of bulliform cells.

In rice a series of genes, such as *ROC5 (RICE OUTER CELL SPECIFIC 5)* and its downstream gene *(PFL) PROTODERMAL FACTOR LIKE* (Zou et al., 2011), *ACL1 (ABAXIALLY CURLED LEAF 1)*, its homologous gene *ACL2* (Li et al., 2010), *ROLLED AND ERECT LEAF 1* (*REL1)* (Chen et al., 2015), *REL2* (Yang et al., 2016), *(LATERAL ORGAN BOUNDARIES DOMAIN 3-7) OsLBD 3-7* (Li C. et al., 2016), *(NARROW LEAF 7) NAL7* (Fujino et al., 2008), *NAL2/3* (Cho et al., 2013), *(BRASSINOSTEROID INSENSITIVE 1-ASSOCIATED KINASE 1) OsI-BAK1* (Khew et al., 2015), and *(AUXIN RESPONSE FACTOR) OsARF18* (Huang et al., 2016) regulate leaf rolling by affecting both number and area of bulliform cells. For example, defects of *ROC5*, a member of *HDZIP CLASS IV* gene family displayed abaxial rolling of leaf. Studies have also reported that reduced or increased expression level of *ROC5* can affect leaf rolling, it serves as a negative regulator leaf rolling by mediating number and size of bulliform cells. Further studies also disclosed *ROC5* probably regulates leaf rolling by binding to L1 motif box in promoter region of *PFL*, as loss-of-function of *PFL* also displayed phenotypes of abaxial leaf rolling (Zou et al., 2011). Higher expression of *ROC5* in *rl* (*t*) mutant revealed positive correlation of bulliform cell area and LRI (leaf rolling index) in rice (Li et al., 2014). *OsARF18* is involved in auxin signaling pathway and is a major target of *miRNA160*. The transgenic plants expressing *miR160-*resistance for *OsARF18* displayed adaxial curling of leaves, due to decreased number and size of bulliform cells (Huang et al., 2016). Similarly, *SLL2 (SHALLOT-LIKE 2)* showed more adaxial curling of leaves in order to increase the photosynthetic activity through regulation of bulliform cells and other related genes (Zhang J.J. et al., 2015). *OsRRK1 (RECEPTOR-LIKE CYTOPLASMIC KINASE 1)* also showed an erect morphology with decreased number and size of bulliform cells in order to get high seed setting (Ma Y. et al., 2017). Keeping in view the functions of above mentioned genes it can be suggested that bulliform cells regulate leaf rolling by changing their size and number through complex interaction of several genes.

### Sclerenchymatous Cells

Rice *SLL1* encodes a transcription factor of *KANADI* family and its loss-of-function mutant showed extreme rolling of leaves phenotype caused by defective development of sclerenchymatous cells on the abaxial side. Further studies exposed *SLL1* regulates leaf rolling by mediating programmed death of sclerenchymatous cells and inhibiting development of abaxial features (Zhang et al., 2009). *SRL2* deficiency also brings about abnormal development of sclerenchymatous cells on the abaxial surface of leaf blade leading to adaxial rolling of leaves. Although, *SLL1* and *SRL2* both were involved in the sclerenchymatous cells development, the analysis of *sll1:srl2* double mutant revealed that these two genes play role in different pathways to regulate leaf rolling (Liu et al., 2016). Moreover, *NRL2* protein interacted with *RL14* and distresses contents of cellulose, lignin and differentiation of sclerenchymatous cells (Zhao et al., 2016). These findings suggested that sclerenchymatous cells also play their part in regulation of leaf rolling.

### Cuticle Development

Histological studies indicated that defective cuticle development is responsible for the rolling of leaves (Wu and Gu, 2011). Molecular mechanism of *CFL1 (CURLY FLAG LEAF 1)* in cuticle development exhibited its involvement in leaf rolling. *CFL1* encodes a WW DOMAIN PROTEIN that was isolated from *cfl1* mutant with curling leaf phenotype. Moreover, overexpression of *OsMYB103L* encodes a transcription factor R2R3MYB that regulates contents of cellulose and mechanical strength of leaves (Yang et al., 2014). It signifies role of cuticle development in developing normal leaf rolling.

### miRNAs

There is a consensus that miRNAs play an important role in leaf development by negatively regulating expression of genes at post-transcriptional level (Moon and Hake, 2011). For example, AGO protein characterized by PAZ and PIWI domains is a core component of RNA-induced silence complex (RISCs) that play crucial role in rice leaf development. Overexpression of *OsAGO7* leads to upward rolling of leaf blade (Shi et al., 2007). Meanwhile, transgenic plants that were knocked down against *AGO1s* via an RNAi silencing approach exhibited pleiotropic defects in phenotypic development, e.g., low height, narrow and rolled leaves (Wu et al., 2009). Additionally, *miRNA160* has been found to be involved in leaf rolling by targeting *OsARF18* (Huang et al., 2016). *HOMEODOMAIN CONTAINING PROTEIN 4 (OsHB4)* is major target of *miRNA166* and play role in development of xylem and transpiration rate. Knockdown of *miRNA16*6 in short tandem target mimic (sttm166) line resulted in rolling of leaf, which had smaller bulliform cells and reduced stomatal conductance (Zhang et al., 2018). The RNaseIII enzyme *DICER-LIKE 1 (DCL1)* is required for the miRNA biogenesis and has pivotal influences on plant growth and development. Down-regulation of *OsDCL1* in RNAi lines leads to a phenotype of narrow leaf blade (Liu et al., 2005). Thus, some miRNAs indirectly can affect the leaf rolling via targeting other genes such as *OsAGO7* and *OsHB4.*

## Role of Genes in Controlling Leaf Size

Leaf size has long been regarded as central agronomic trait in rice and received tremendous attention. A range of QTLs for leaf size have been identified on several chromosomes in rice and most of genes governing leaf size were mainly identified through recessive mutations. Leaf size is regulated by different genes through various internal and external features of plant and environment, which are described below.

### Number of Veins

Grasses leaves are of mostly strap shape, in which veins run in parallel fashion from base to tip and distance among the veins create the differentiation of C3 from C4 plants (Wang et al., 2017). The vascular system found in veins is thought to be directly associated with leaf size. *NAL1* encodes a plant specific protein and preferentially expressed in vascular tissues. Reduced expression of *NAL1* leads to decreased number of leaf veins that might be responsible for the narrow leaf phenotype (Qi et al., 2008). Besides of reduced expression, recessive mutation in *NRL1* resulted in narrow leaves with reduced number of veins (Jiang et al., 2010). *NAL2* and *NAL3* (*NAL2/3*) encode WUSCHEL-RELATED HOMEOBOX PROTEIN in rice that are duplicate orthologs of genes *NS1 (NARROW SHEATH 1)* and *NS2* in maize, respectively. The *nal2:nal3* double mutant leaves contains decreased number of veins with extremely narrow leaves (Cho et al., 2013). Similarly, *NAL9 and NRL2* also known as *ClpP and SRL2*, respectively, regulate leaf size through affecting the number of veins (Dong H. et al., 2013; Li et al., 2013). *OsaMIR319a* or *Osa-MIR319b* overexpression, both results in wider leaves, owing to an increased number of longitudinal small veins, suggesting miRNA*s* also regulate leaf size in rice (Yang et al., 2013).

Fourteen QTLs for flag leaf length and nine for breadth were detected in chromosomal substitution line (CSSL) in rice and candidate gene for *qFW4-2* was *NAL1* (Tang et al., 2018). Yield per plant and flag leaf width have positive association found in *qFLW7.2* and *qPY7* and both of them were positioned on chromosome 7 (Zhang B. et al., 2015). These evidences support the idea that leaf size had an obvious positive correlation with number of leaf veins.

### Cell Division

*NAL1*, *NRL1*, and *AVB (ABNORMAL VASCULAR BUNDLES)* all are reported to be involved in cell division, implying these genes probably affect cell division to regulate leaf size (Jiang et al., 2015; Ma L. et al., 2017). *OsCCC1*, a member of *CATION-CHLORIDE CO-TRANSPORTER* family localized in plasma membrane played its role in ionic transportation. Recent finding demonstrates that *OsCCC1* participates in elongation of cells by mediating ionic (*K*^+^*, Cl*^-^, and *Na*^+^) homeostasis to sustain osmoregulation, and loss-of- function of *OsCCC1* leads to narrow leaves illustrating cell division also accounts for narrow leaves phenotype (Kong et al., 2011; Zhi et al., 2016). Additionally, *PLA1 (PLASTOCHRON 1)* and *PLA2* encode cytochrome *P450* and an RNA*-*binding protein, respectively, and both of them were reported to regulate leaf size mainly due to increase in cell size (Miyoshi et al., 2004). Two QTLs, *qTSN4* and *qTSN12* found in their NILs revealed, additional leaf area was produced by oversizing of meristems (Adriani et al., 2016). *OsGASR1 (GA-STIMULATED RICE 1)* that belongs to family of *GAST* [*GIBBERELLIN (GA)-STIMULATED TRANSCRIPT*] showed higher expression in regions of cell proliferation and increase leaf blade size due to increase in cell length. Its mRNA expression could be triggered by an exogenous application of gibberellins (Lee et al., 2017). *OsGIF1 (GRF-INTERACTING FACTOR 1)* influenced grain production and size of leaves in rice by regulating leaf cell size (He et al., 2017). *OsGIF1* positively regulates cell proliferation and revealed conserved functional control of *MAKIBA3* (*MKB3*) and *ANGUSTIFOLIA3* (*AN3*) in rice and Arabidopsis, respectively. A loss-of-function mutant *MKB3* exhibited narrowed- and rolled-leaf phenotype (Shimano et al., 2018). Overexpression of an α-expansin gene *OsEXPA8* (*EXPANSIN 8*) produced improved root system, enhanced leaf number and enlarged leaf size in rice. Further analysis of *OsEXPA8* line showed increased lignin content in cell wall and enhanced length of leaf cells (Ma et al., 2013). Expression of *OsEBS (ENHANCING BIOMASS AND SPIKELET NUMBER*) in rice caused increase in plant height, leaf size and spikelet number per panicle due to increase in cell number (Dong X. et al., 2013). A semi-dwarf mutant in which a single copy of transposon *dissociator* (*Ds*) was inserted into gene *OsCYP96B4* (*CYTOCHROME P450 96B4*). It showed defects in plant height and length of leaf sheath cells (Ramamoorthy et al., 2011). So, keeping in view function of these genes in cell division (i.e., size and number), their use in breeding can provide an effective tool to engineer plants with more leaf area for better agronomic yield.

### Phytohormones

Plant hormones such as auxin and gibberellins are necessary for plant development and play a substantial role in regulation of leaf size. Several genes have been characterized and involved in biosynthesis, transport and signal transduction pathways of some phytohormones. *OsCOW1 (CONSTITUTIVELY WILTED 1)* gene is identical to *NAL7* that encodes a FLAVIN-CONTAINING MONO-OXYGENASE protein and indicated resemblance with *YUCCA* in Arabidopsis and *FLOOZY* in petunia, which encode for auxin biosynthesis (Woo et al., 2007). Overexpression and knock-down of *OsCOW1* formed wide and narrow leaves, respectively. Further studies have also disclosed function of some members of *YUCCA* gene family that were involved in a tryptophan-dependent IAA biosynthetic pathway of rice and Arabidopsis (Gallavotti et al., 2008). Physiological and quantitative real-time PCR analysis of *OsPIN1 (PIN-FORMED 1)* showed that it can serve as an auxin efflux facilitator (Xu et al., 2005). *NAL1* regulates polar transport of auxin in rice by *OsPIN1* (Jiang et al., 2015). Another, leaf size gene *NAL2/3* also found to be involved in distribution of auxin. In addition, *TDD1 (TRYPTOPHAN DEFICIENT DWARF MUTANT 1)* and *FIB (FISH BONE)* both were involved in auxin biosynthesis and mutants with reduced expression of *TDD1* or *FIB* showed narrow leaves phenotype (Sazuka et al., 2009; Yoshikawa et al., 2014). Overexpression of *OsARF19* (Zhang S. et al., 2015) or its downstream gene *OsGH3-5*, also revealed narrow leaves phenotype due to changes in level of phytohormones (Zhang S. et al., 2015).

Another class of phytohormones, gibberellin has proved to be involved in leaf expansion. A recent study showed that *NAL2/3* not only regulate distribution of auxin but also provide negative feedback to gibberellin biosynthesis for gibberellin homeostasis in rice. It is indicating that *NAL2/3* probably regulate leaf size via the crosstalk of GA and auxin (Cho and Paek, 2016). Rice *PLA1* and *PLA2* genes act downstream in gibberellin signal transduction pathway and their loss-of-function caused shortening of leaves (Mimura et al., 2012). In contrast, the mutant disrupted in *SLR1* that is a negative regulatory factor for the gibberellin signal transduction, displayed elongated leaves in rice. Phenotypes of *pla1* and *pla2* mutants displayed rapid leaf emergence and small organs, and found gibberellin is the major phytohormone associated with *PLA1* and *PLA2* functions. Both, *PLA1* and *PLA2* act downstream of GA signal transduction pathway to regulate leaf development (Mimura et al., 2012). Catabolism of double mutants indicated that *PLA1* and *PLA2* were partially necessary for leaf elongation depending on the gibberellin contents. Moreover, altered expression of *(OVATE FAMILY PROTEIN 2) OsOFP2* (Schmitz et al., 2015), *OsGA2ox6* (Huang et al., 2010), *(DWARF 1*) *D1* (Ashikari et al., 1999), and *(GIBBERELLIN-INSENSITIVE DWARF 2) GID2* (Sasaki et al., 2003) all were involved in regulation of gibberellin pathway and causes changes in leaf size. Besides auxin and gibberellin altered leaf size was also observed in mutants that were driven by different genes involved in regulation of other phytohormones. However, little is known at present whether or how extent the leaf size is associated with other phytohormones.

Interestingly, in most cases the genes controlling leaf size also have an effect on plant height. For example *NAL1* regulates both plant height and leaf size. *DNL1 (DWARF AND NARROW LEAF 1)* allelic to *NRL1*, is a QTL for leaf size and plant height (Ding et al., 2015). Compared to wild type, mutant showing loss-of-function of *SLL1* gene displayed narrow rolled leaves and reduced plant height (Zhang et al., 2009). Although, number of other changes were also observed in plants showing reduced activity of *NAL2/3*. Overexpression of *NAL2/3* leads to dwarf phenotype in rice (Cho et al., 2013). However, the leaf size doesn’t have positive correlation with plant height, for example, *dwarf 1* (*d1)* mutant have wider leaves phenotype and overexpression of *SG1 (SHORT GRAIN 1)* results in reduced plant height and increased leaf size (Nakagawa et al., 2012). Suppression of *miR159* controls plant height and leaf length in *sttm159* transgenic plants by expression of those genes that were involved in phytohormones homeostasis (Zhao et al., 2017). Study of *osgasr1* mutant suggested that *OsGASR1* played important roles in expression of α-amylase gene and regulate growth of seedling by increasing cell length (Lee et al., 2017). This anomalous relationship between leaf size and plant height needs further investigations.

### Epigenetic Aspects

Histone modifications are important part of epigenetic mechanisms and have determinant role in controlling leaf size. Different stresses act as stimulus and change the genic expression level by various epigenetic mechanisms, e.g., DNA methylation, histone modifications and miRNA (Saraswat et al., 2017). *HDT702* is a member of the *HISTONE DEACETYLASE (HDAC)* gene family that plays an important function in histone modifications and plant gene expression. Down-regulation of *HDT702* results in narrow leaves, indicating that histone modifications are also involved in regulation of leaf size (Hu et al., 2009).

## Conclusion

**FIGURE 1:**
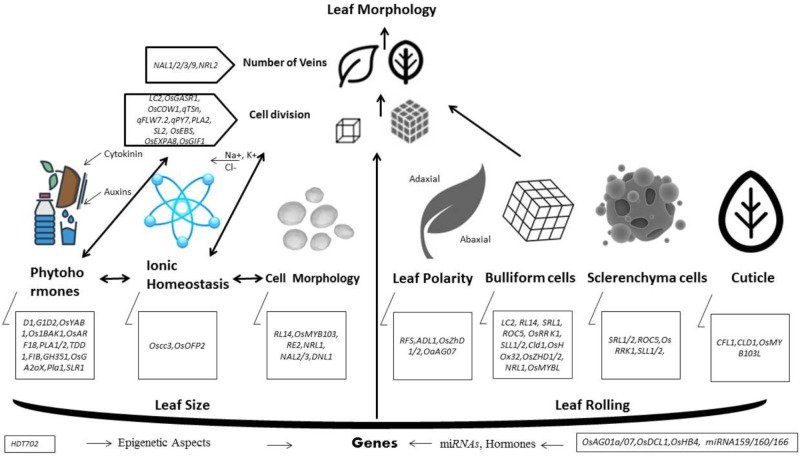
A hypothetical model to understand working of genes regulating leaf morphology.

In past several years, molecular and genomic studies disclosed important advancements in identification of genes or QTLs controlling leaf morphology (Table 1). Innovative cloning and sequencing technologies has made possible to identify new genes much easier and faster than before. In order to conclude our understanding, we built a hypothetical working model (Figure 1) that is showing a logical mechanistic control of leaf morphology. According to this model, different genes involved in leaf polarity, bulliform cells, sclerenchyma cells and cuticle development exhibit the final phenotype of leaf rolling. While, leaf size is mainly controlled by numerous genes encoding phytohormones, cell division, number of veins, ionic homeostasis and epigenetic aspects. Defective development of cell wall formation, impaired cell division, abnormal contents of cellulose and lignin, reduced water retaining capacity, failure of developing homeostasis in cellular structures of leaf, and poor development of sclerenchyma cells on either side of leaf blade are main causes of leaf rolling. Leaf size genes are partially obsessed or affected by epigenetic mechanisms, ionic homeostasis, number of veins in leaf, cell division and regulation of phytohormones.

**Table 1:** List of rice genes controlling leaf rolling and size.

Gene symbol	Gene product	Function	Reference
*ACL1* and *ACL2*	Unknown protein	Function in leaf development	Li et al., 2010
*ADL1*	CALPAIN-LIKE CYSTEINE PROTEASE	Involved in establishment of the adaxial–abaxial axis	Hibara et al., 2009
*AVB*	Plant conserved protein with unknown functions	Leaf cell number via auxin regulation	Ma Y. et al., 2017
*CFL1*	Transcription factor	Regulate cuticle development	Wu and Gu, 2011
*CLD1/SRL1/2*	GLYCOSYLPHOSPHATIDYL-INOSITOL protein	Cell wall integrity and osmotic homeostasis	Xiang et al., 2012; Liu et al., 2016; Li et al., 2017
*D1*	A-subunit of GTP-BINDING PROTEIN	Function in gibberellin signal transduction	Ashikari et al., 1999
*DNL1*	CELLULOSE SYNTHASE-LIKE D4	Controls leaf width	Ding et al., 2015
*FIB*	TRYPTOPHAN AMINOTRANSFERASE	Involved in auxin biosynthesis	Yoshikawa et al., 2014
*GH3-5*	INDOLE-3-ACETIC ACID-AMIDOSYNTHETASE	Involved in phytochrome and Jasmonate signaling	Zhang S. et al., 2015
*GID2*	An F-BOX PROTEIN	Function in gibberellin signal transduction	Sasaki et al., 2003
*HDT702*	HISTONE DEACETYLASE	Involved in Histone modifications	Hu et al., 2009
*LC2*	VIN3-LIKE PROTEIN	Function in cell division	Zhao et al., 2010
*MKB3*	SNH (SYT N-TERMINAL HOMOLOGY) domain	Proliferation of leaf cell	Kim and Tsukaya, 2015
*miRNA159*	Expression of *OsGAMYB* and *OsGAMYBL 1*	Control length of flag leaf by phytohormones	Zhao et al., 2017
*NAL1*	TRYPSIN-LIKE SERINE and CYSTEINE PROTEASE	Regulate vein patterning and polar auxin transport	Qi et al., 2008
*NAL2/3*	WUSCHEL-RELATED HOMEOBOX PROTEIN	Affect leaf margin development and vascular patterning	Cho and Paek, 2016
*NAL7*	FLAVIN-CONTAINING MONOOXYGENASE	Leaf shape mediated by auxin	Fujino et al., 2008
*NAL9*	ATP-DEPENDENT CLP PROTEASE	Involved in leaf development	Dong H. et al., 2013
*NRL1*	CELLULOSE SYNTHASE-LIKE PROTEIN D4	Regulate cell wall formation	Hu et al., 2010
*NRL2*	A novel protein with unknown biochemical function	Leaf shape	Zhao et al., 2016
*OsAGO1a*	ARGONATUE PROTEIN	Form miRNA effector complexes	Li et al., 2013
*OsAGO7*	ARGONATUE PROTEIN	Form miRNA effector complexes	Shi et al., 2007
*OsCYP96B4*	*CYTOCHROME P450*	Involved in length of leaf sheath cells	Ramamoorthy et al., 2011
*OsEXPA8*	Cell wall EXPANSINS	Increase leaf number and size by cell expansion	Ma et al., 2013
*Osa-MIR319a, Osa-MIR319b*	Expression of *mi*319	Target *TCP* genes (*OsPCF5* and *OsPCF8)* and control leaf width	Yang et al., 2013
*OsARF18/19*	AUXIN RESPONSE FACTOR/target of *miRNA160*	Involved in auxin signaling	Huang et al., 2016; Zhang S. et al., 2015
*OsCCC1*	A putative CATION-CHLORIDE COTRANSPORTER	Functions as a K^+^, Na^+^Cl^-^cotransporter	Chen et al., 2015
*OsCOW1*	FLAVIN-CONTAINING MONOOXYGENASE	Control the development of leaf width	Woo et al., 2007; Gallavotti et al., 2008
*OsDCL1*	miRNA factor	Growth defects	Liu et al., 2005
*OsEBS*	*qGP5-1*	Increase leaf size by cell number	Huang et al., 2010
*OsGA2ox6*	GIBBERELLIN 2-OXIDASE	Function in the gibberellin catabolic pathway	Huang et al., 2010
*OsGASR1*	GAST [GIBBERELLIN (GA)-STIMULATED TRANSCRIPT] FAMILY	Increase leaf size by cell division	Lee et al., 2017
*OsGIF1*	GROWTH REGULATING FACTOR-INTERACTING FACTOR 1	Increase leaf size by cell size	He et al., 2017
*OsHB4*	Target of *Osa-miR166*	Size of bulliform and sclerenchymatous cells	Zhang et al., 2018
*OsHox32*	An HD-ZIP III FAMILY PROTEIN	Functions in leaf development	Li Y.Y. et al., 2016
*OsPIN*	PIN-FORMED 1 Protein	Auxin-dependent regulation of shoot	Xu et al., 2005
*OsI-BAK1*	BRASSINOSTEROID INSENSITIVE 1-ASSOCIATED KINASE I	Involved in BR signaling pathway	Khew et al., 2015
*OsLBD3-7*	DUF260 DOMAIN CONTAINING PROTEIN	Works as a transcription activator	Li C. et al., 2016
*OsMYB103L*	An R2R3-MYB TRANSCRIPTION FACTOR	Mediates cellulose biosynthesis and secondary walls formation	Yang et al., 2014
*OsOFP2*	OVATE FAMILY PROTEIN	Affect hormonal homeostasis and vascular development	Schmitz et al., 2015
*OsRRK1*	RECEPTOR-LIKE CYTOPLASMIC KINASE	Size and number of bulliform cells	Ma Y. et al., 2017
*OsYAB1*	YABBY DOMAIN CONTAINING PROTEIN	Involved in gibberellin metabolism	Dai et al., 2007
*OsZHD, OsZHD2*	Transcription factor	Involved in abaxially curling and drooping of leaf in rice	Xu et al., 2014
*PFL*	PROTODERMAL FACTOR LIKE PROTEIN	Regulation of bulliform cells	Zou et al., 2011
*PLA1*	CYTOCHROME P450, CYP78A11	Regulate leaf growth downstream of the GA pathway	Mimura et al., 2012
*PLA2*	An RNA-BINDING PROTEIN	Regulate leaf growth downstream of the GA pathway	Mimura et al., 2012
*qFLW7.2* and *qPY7*	*LOC_Os07g41180* and *LOC_Os07g41200*	Controls flag leaf length and width	Zhang B. et al., 2015
*qFW4-2*	Regulate *NAL1* candidate gene	Controls flag leaf length and width	Tang et al., 2018
*qTSN*	QTLs^∗^	Meristem oversizing	Adriani et al., 2016
*REL1*	A novel unknown protein	Positively regulate leaf rolling and bending	Chen et al., 2015
*REL2*	DUF630 and DUF632 DOMAINS PROTEINS	Function in the leaf shape formation	Yang et al., 2016
*RFS*	CHD3/MI-2	Control leaf polarity by epigenetic factor	Cho et al., 2018
*Rl* (t)	HD-GL2 (HOMEODOMAIN-GLABRA2)	Controls leaf rolling in a dosage-dependent manner	Li et al., 2014
*RL14*	2OG-FE (II) OXYGENASE FAMILY PROTEIN	Regulate secondary cell wall formation	Fang et al., 2012
*ROC5*	HOMEODOMAIN LEUCINE ZIPPER CLASS IV PROTEIN	Negatively regulates bulliform cell fate	Zou et al., 2011
*SLL1*	SHAQKYF CLASS MYB FAMILY transcription factor	Regulate sclerenchyma cell development	Zhang et al., 2009
*SLL2*	T-DNA insertion in *LOC_Os07g38664*	Regulation of bulliform cells	Zhang J.J. et al., 2015
*TDD1*	ANTHRANILATE SYNTHASE B-SUBUNIT	Functions upstream of Trp-dependent IAA biosynthesis	Sazuka et al., 2009

^∗^*QTLs are not gene product*.

Biotic and a-biotic stresses such as high temperature, drought, fungus and insects cause severe yield losses to plants (Kadioglu et al., 2012). Plant makes adjustments in leaf morphology to avoid or compensate the effect of those stresses by gene regulation and plant metabolism. Genes that are controlling the ideal morphology of leaf, and enable the plant to cope challenges of changing climate and environment will be an important area of future research. Finding of physiological and molecular mechanisms underlying genes that are governing desired characteristics would be beneficial in wheat, rice, maize and other crops. Knowledge of cause of changes in morphology and mechanism determining these changes may lead in way to develop better strategies in molecular breeding. Keeping in view of this need, this review can provide an umbrella of genes function to understand leaf morphogenesis of monocot especially in rice. We can utilize these gene in molecular breeding of rice in order to improve grain yield by gaining advantages of ideal leaf morphology. In spite of great progress, some areas still need further research, e.g., which leaf morphology associated genes are under epigenetic control? Which other factors are playing role in upstream of signaling pathways that are regulating leaf morphology. Is the regulation of leaf morphology is conserved in all plant species?

## Author Contributions

PX and AA wrote the review. XW read and approved the contents. BH helped in literature and reference digestion.

## Conflict of Interest Statement

The authors declare that the research was conducted in the absence of any commercial or financial relationships that could be construed as a potential conflict of interest.

## Footnotes

1 http://www-plb.ucdavis.edu/labs/rost/Rice/Leaves/internal.html
